# Vascular Surgery in Japan: 2014 Annual Report by the Japanese Society for Vascular Surgery

**DOI:** 10.3400/avd.ar.20-00128

**Published:** 2020-12-25

**Authors:** The Japanese Society for Vascular Surgery Database Management Committee Member, NCD Vascular Surgery Data Analysis Team

**Keywords:** peripheral arterial disease, stent graft, endovascular treatment, aneurysm, varicose vein treatment

## Abstract

**Objectives**: This is an annual report indicating the number and early clinical results of annual vascular treatment performed by vascular surgeon in Japan in 2014, as analyzed by database management committee (DBC) members of the JSVS.

**Materials and Methods**: To survey the current status of vascular treatments performed by vascular surgeons in Japan, the DBC members of the JSVS analyzed the vascular treatment data provided by the National Clinical Database (NCD), including the number of treatments and early results such as operative and hospital mortality.

**Results**: In total 113,296 vascular treatments were registered by 1,002 institutions in 2014. This database is composed of 7 fields including treatment of aneurysms, chronic arterial occlusive disease, acute arterial occlusive disease, vascular injury, complication of previous vascular reconstruction, venous diseases, and other vascular treatments. The number of vascular treatments in each field was 21,085, 14,344, 4,799, 2,088, 1,598, 42,864, and 26,518, respectively. In the field of aneurysm treatment, 17,973 cases of abdominal aortic aneurysm (AAA) including common iliac aneurysm were registered, and 55.7% were treated by endovascular aneurysm repair (EVAR). Among AAA cases, 1,824 (10.1%) cases were registered as ruptured AAA. The operative mortality of ruptured and un-ruptured AAA was 16.1%, and 0.6%, respectively. 32.1% of ruptured AAA were treated by EVAR, and the EVAR ratio was gradually increasing, but the operative mortality of open repair and EVAR for ruptured AAA was 15.7%, and 18.0%, respectively. Regarding chronic arterial occlusive disease, open repair was performed in 8,020 cases, including 1,210 distal bypasses to the crural or pedal artery, whereas endovascular treatment (EVT) were performed in 6,324 cases. The EVT ratio was gradually increased at 44.1%. Venous treatment including 41,246 cases with varicose vein treatments and 520 cases with lower limb deep vein thrombosis were registered. Regarding other vascular operations, 25,024 cases of vascular access operations and 1,322 lower limb amputation surgeries were included.

**Conclusions**: The number of vascular treatments increased since 2011, and the proportion of endovascular procedures increased in almost all field of vascular diseases, especially EVAR for AAA, EVT for chronic arterial occlusive disease, and endovenous laser ablation (EVLA) for varicose veins. (This is a translation of Jpn J Vasc Surg 2020; 29: 15–31.)

## Introduction

In 2011, the National Clinical Database (NCD) was launched, and registration of surgical cases commenced in the same year. The Japanese Society for Vascular Surgery (JSVS) uses this database to tabulate vascular surgeries published annually in a vascular surgery conference.^1)^ In this paper, we report the results obtained for vascular surgery cases registered in the NCD from January to December 2014. Members of the JSVS Database Management Committee collected and analyzed data from this database.

## Methods

After approval from JSVS (a governing society of the NCD), we extracted data pertaining to vascular surgeries registered in 2014 in the NCD. The data was classified into seven categories, tabulated, reviewed, and analyzed by members of the JSVS Database Management Committee. The categories include revascularization for aneurysms, revascularization for chronic arterial occlusion, revascularization for acute arterial occlusion, treatment for vascular trauma, surgery for vascular complications after revascularization, venous surgery, and other vascular disease and related surgery.

The tabulation results present the number of cases according to the different surgical procedures, the underlying pathology, operative mortality, in-hospital mortality, and the materials used. Operative mortality (which is synonymous with surgery-related death), includes all deaths within 30 days of surgery, irrespective of the cause or whether the patient died during hospitalization. Furthermore, in-hospital mortality was defined as death occurring at any time during the same hospital stay as the surgery.

Some discrepancies are present in the table. For example, the total number of underlying pathologies and materials used are inconsistent with the total number of cases. However, after careful investigation, the JSVS Database Management Committee and NCD concluded that the discrepancies were attributable to four factors. These include permission of multiple choices, when no choice was permitted, omissions or incorrect input by the data entry operator, and instances of multiple materials being used or multiple sites being treated for a single case. As from 2013, countermeasures have been taken to prevent data entry errors as much as possible. These measures include laying out or creating new choices, and modifying the program, wherever possible, to avoid blank fields and omissions in the registration form.

**Table 1** displays the modified items in the registration or tabulation method in 2014.

**Table:** Table 1 New items or changes in 2014 annual report

New items	Table number	status until 2013
Previous reconstruction	Tables 3-1, 3-2, 3-3, 3-4, 3-5	Not existed
None	Tables 3-1, 3-2, 3-3, 3-4, 3-5	Not existed
Once	Tables 3-1, 3-2, 3-3, 3-4, 3-5	Not existed
Twice	Tables 3-1, 3-2, 3-3, 3-4, 3-5	Not existed
Three times and more	Tables 3-1, 3-2, 3-3, 3-4, 3-5	Not existed
Unclear	Tables 3-1, 3-2, 3-3, 3-4, 3-5	Not existed
Revision site	Tables 3-1, 3-2, 3-3, 3-4, 3-5	Not existed
Host artery stenosis/occlusion	Tables 3-1, 3-2, 3-3, 3-4, 3-5	Not existed
Graft/EVT stenosis	Tables 3-1, 3-2, 3-3, 3-4, 3-5	Not existed
Graft/EVT occlusion	Tables 3-1, 3-2, 3-3, 3-4, 3-5	Not existed
Others	Tables 3-1, 3-2, 3-3, 3-4, 3-5	Not existed
Unclear	Tables 3-1, 3-2, 3-3, 3-4, 3-5	Not existed

## Tabulation/Statistical Analysis Results

In 2014, 113,296 vascular surgery cases were registered in the NCD (an increase of 12.8% over the previous year), exceeding 110,000 cases. This accounted for 8.7% of all registered surgeries for the same year. Furthermore, vascular surgeries were registered from 1,002 institutions. Thus, 29.1% of the registering institutions were for vascular surgeries. Moreover, of those 1,002 institutions in 2014, 413 institutions (41.2%) were certified cardiovascular surgery training centers that contributed to the registration of this data. The tabulation results of 2014 for each category are presented below. Furthermore, we performed statistical analysis using a chi-square test, and p values <0.05 were considered statistically significant.

## 1. Treatment for Aneurysm (Table 2)

### 1) Thoracic aortic aneurysms

Most cases of surgery for thoracic aortic aneurysms are registered in the JCVSD, whereas those performed by vascular surgeons are tabulated in the NCD (**Table 2**). Therefore, at present, surgeries for thoracic aortic aneurysms performed throughout Japan are registered in a fragmented manner. This makes it impossible to obtain an accurate overall image of the current state. In the future, we recommend that these organizations come together, in order to facilitate the understanding of the status quo of surgery for thoracic aortic aneurysms nationwide.

**Table:** Table 2 Treatment for aneurysm Table 2-1 Aortic aneurysm

Region of aortic aneurysm	Cases	Gender		Mortality		Ruptured aneurysm			Dissection*^3)^	Etiology							
		Male	Female	30-day mortality	Hospital mortality	Cases	30-day mortality	Hospital mortality		Degenerative*^4)^			inflammatory	Vasculitis	Infected	Connective tissue disease*^5)^	Others
										Cases	30-day mortality	Hospital mortality					
Ascending aorta*^1)^	98	65	33	14	16	14	6	7	57	88	13	15	0	0	2	0	8
Aortic arch*^1)^	514	402	112	24	33	49	6	11	158	466	21	28	1	0	10	14	23
Descending thoracic aorta*^1)^	483	361	122	24	33	92	15	23	179	412	17	22	6	0	20	13	32
Thoracoabdominal aorta*^1)^	369	281	88	27	40	58	14	21	121	319	21	31	4	2	13	10	21
Abdominal aortic aneurysm*^2)^	17,973	14,897	3,075	390	507	1,824	293	341	684	17,132	360	463	246	17	310	23	245
with renal artery reconstruction	317	273	44	6	11	41	3	5	36	296	6	10	3	0	12	1	5
with renal artery clamping	1,288	1,101	187	51	64	211	33	42	86	1,198	48	59	29	1	38	2	20

＊1) These data are not including cases recorded in JCVSD Database in which most cardiac surgeons were entering their cases. ＊2) Including common iliac artery aneurysm. ＊3) Including both acute and chronic aortic dissection. ＊4) Most likely atherosclerosis. ＊5) Connective tissue abnormalities such as Marfan syndrome.

**Table:** Table 2-1 Aortic aneurysm (continued)

Region of aortic aneurysm	Treatment procedure						Graft materials *^7)^		
	Replacement			Exclusion with bypass	Stent graft	Hybrid *^6)^	Polyester	ePTFE	Others
	Cases	Y-graft	T-graft						
Ascending aorta *^1)^	3	0	0	4	9	5	48	15	9
Aortic arch *^1)^	2	0	0	1	272	165	111	71	9
Descending thoracic aorta *^1)^	7	0	0	1	426	28	30	15	4
Thoracoabdominal aorta *^1)^	22	0	0	8	220	31	104	16	13
Abdominal aortic aneurysm *^2)^	7,967	5,870	1,191	68	9,975	38	7,058	353	79
with renal artery reconstruction	293	216	50	6	8	7	282	29	3
with renal artery clamping	1,258	961	238	9	12	7	1,230	50	5

＊6) Debranch bypass surgery combined with two staged TEVAR is counted as one case of hybrid treatment. ＊7) Only for open surgery.

**Table:** Table 2-2 Abdominal aortic aneurysm mortality classified by treatment procedures

Procedure for aneurysm repair	Ruptured aneurysm			Non-ruptured aneurysm		
	Cases	30-day mortality	Hospital mortality	Cases	30-day mortality	Hospital mortality
Replacement	1,243	195	224	6,724	54	85
Exclusion with bypass	21	4	6	47	2	3
EVAR *^8)^	581	99	118	9,410	41	80
Hybrid	5	1	1	33	0	0

＊8) Abbreviation; EVAR: endovascular aneurysm repair

### 2) Abdominal aorta aneurysm (Tables 2-1 and 2-2)

**Figure 1:**
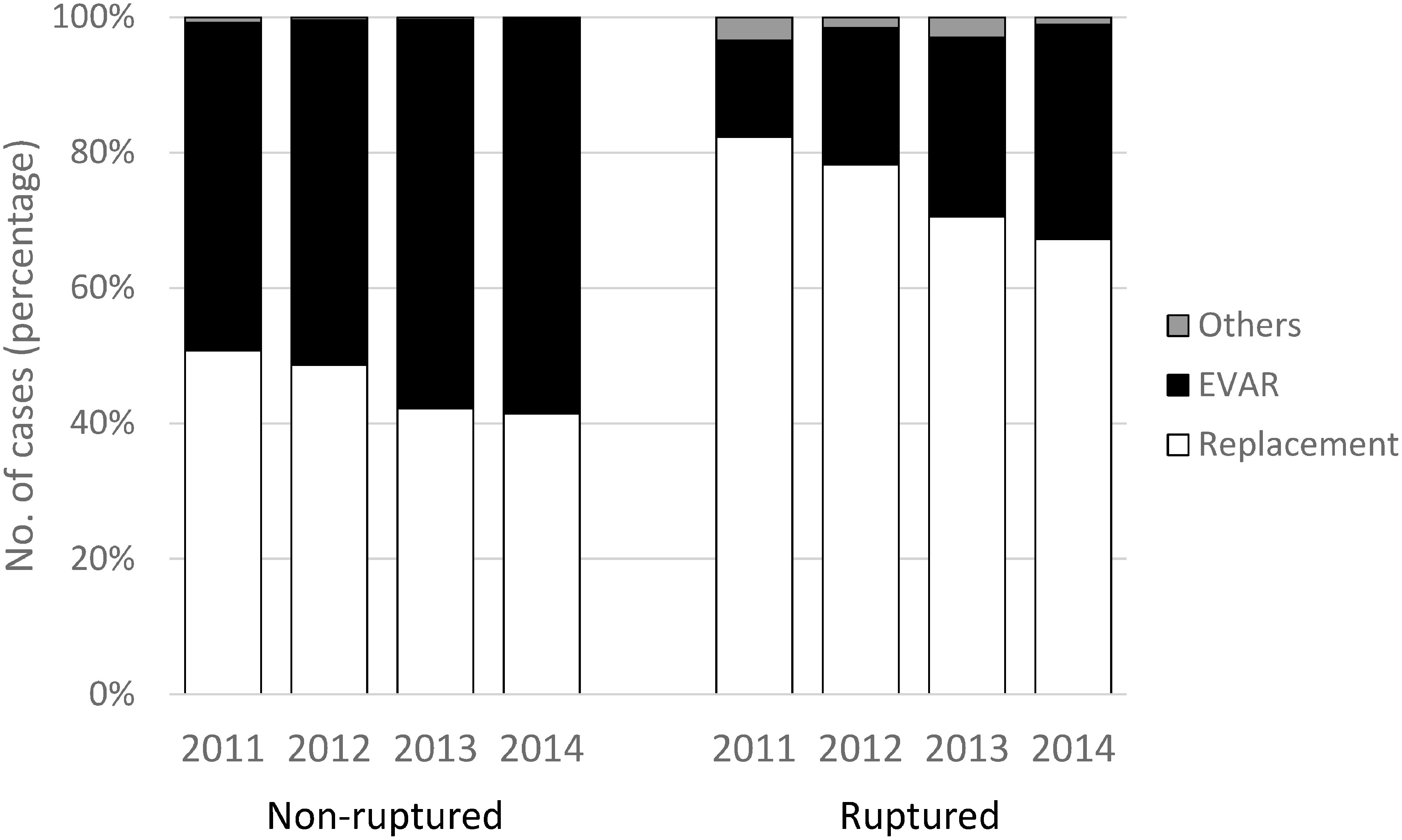
Fig. 1 Treatment procedure for non-ruptured and ruptured abdominal aortic aneurysm (AAA). Comparing year 2011, 2012 and 2013, proportion of EVAR selection was gradually increased in 2014.

In 2014, 17,973 cases of surgery for abdominal aortic aneurysms (including iliac artery aneurysms) were registered in the NCD. This continues to increase by approximately 1,000 cases each year; 15,745 cases in 2012, to 16,694 cases in 2013. Among these cases, replacement surgery was performed in 7,967 cases (44.3%), and stent grafting (endovascular aortic aneurysm repair [EVAR] including hybrid surgery) in 10,013 cases (55.7%) accounting for more than 50% in the previous year, and the rate of which increased further this year (47.6% in 2012, and 52.9% in 2013) (**Fig. 1**). Compared to 2012, the number of replacement surgeries decreased by nearly 1,000 in 2013. However, present data reveals an increasing tendency. This could be attributed to a greater increase in the total number of cases (the denominator) leading to the observed ratio.

Amongst the replacement surgery cases, renal clamping was performed in 1,258 cases (15.8%), and renal artery reconstruction in 293 cases (3.7%). With the evolution of EVAR, an expected increase in the number of cases of pararenal abdominal aortic aneurysms requiring renal artery clamping is envisaged. However, we have not observed a major change despite seven years from the introduction of EVAR.

**Figure 2:**
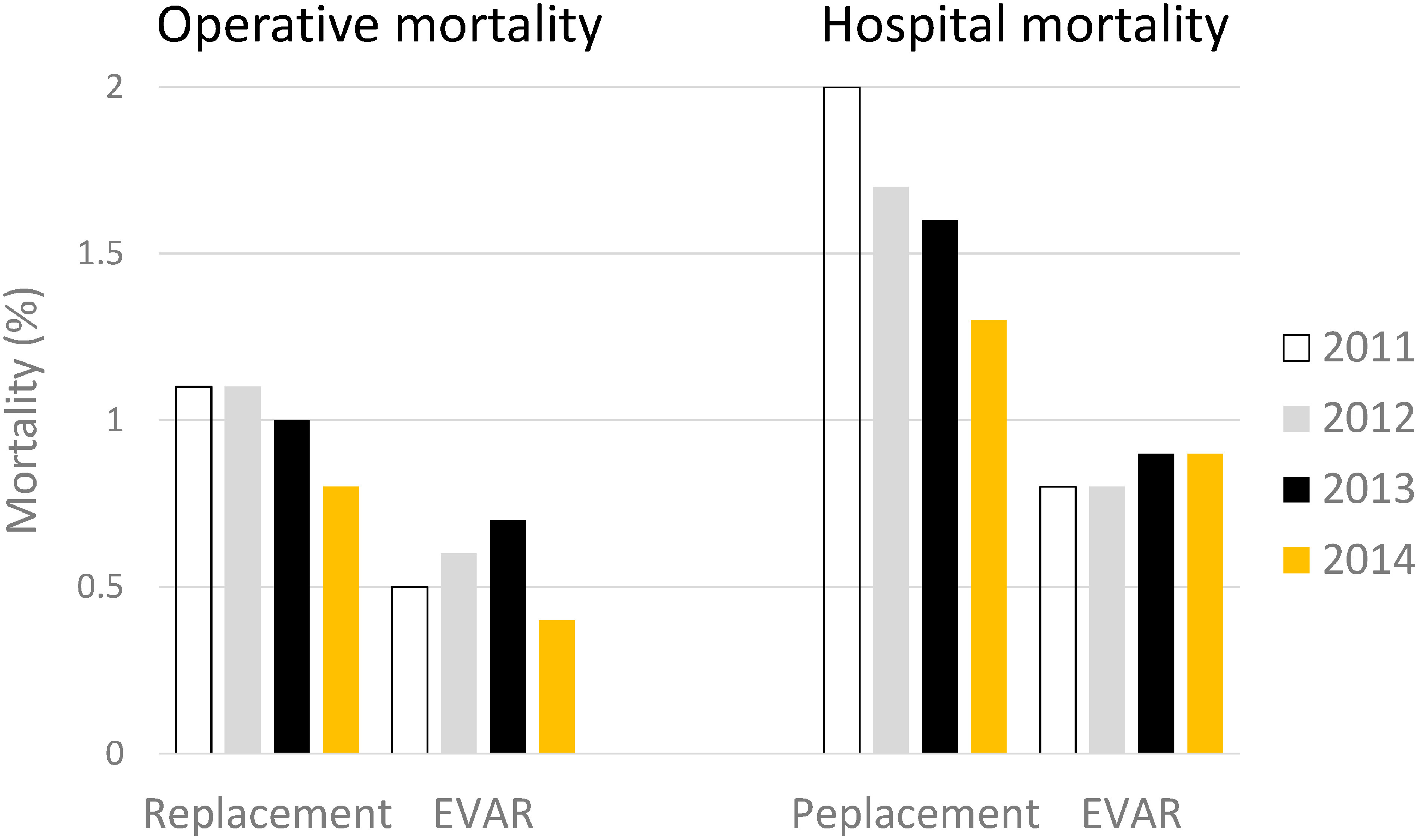
Fig. 2 Early clinical results of non-ruptured AAA in year 2014 comparing with those in year 2011, 2012 and 2013. Regarding the statistical difference of mortality rates between open repair (replacement) and EVAR, see main text.

The operative and in-hospital mortality rates after replacement surgery were 0.8% and 1.3%, respectively, while the corresponding rates after EVAR (including special and hybrid procedures) were 0.4%, and 0.8% respectively (operative mortality: p<0.005, and in-hospital mortality: p<0.05) (**Fig. 2**). Among the replacement surgery cases, the rates were respectively 1.7% and 2.1% higher in those with renal artery clamping, similar to cases requiring reconstruction (1.2% and 2.4%).

EVAR: endovascular aneurysm repair

**Figure 3:**
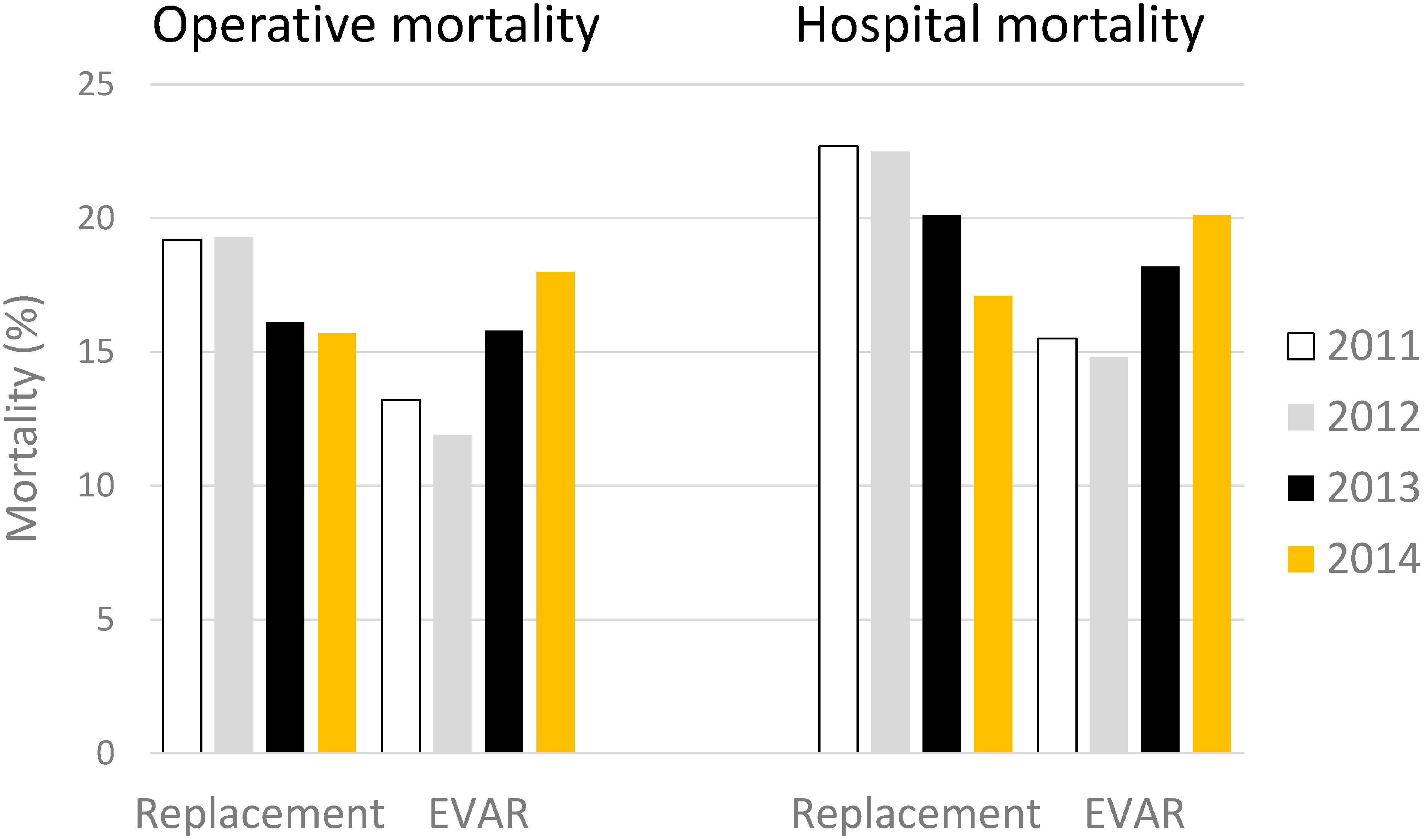
Fig. 3 Early clinical results of ruptured AAA in year 2014 comparing with those in year 2011, 2012 and 2013. Regarding the statistical difference of mortality rates between open repair (replacement) and EVAR, see main text.

We recorded 1,824 cases of surgery for ruptured cases, with an operative mortality was 16.1%, and the in-hospital mortality was 18.7%. Compared to 2013 (17.9% and 21.4%, respectively), a slight improvement was observed. EVAR was performed in 586 cases (32.1%), indicating that EVAR continues to be used at an increasing proportion for cases with rupture (14% in 2011, 20% in 2012, and 25.5% in 2013). The operative mortality and in-hospital mortality rates after EVAR for ruptured cases were 17.1% and 20.3%, respectively, which had deteriorated from 2012 (11.9% and 14.8%, respectively), and 2013 (15.8% and 18.2%, respectively), which is thought to be attributed to the fact that EVAR is now selected for anatomically, and hemodynamically difficult cases. However, we believe that the introduction of EVAR might contribute to improved treatment outcomes for ruptured cases overall (**Fig. 3**).

### 3) Peripheral aneurysm (Table 2-3)

We recorded 1,869 cases, with a male-to-female ratio of 1,383 : 486, indicating a higher incidence in men. The most affected sites were the abdominal visceral arteries (noted in 767 cases), lower limb arteries (717 cases), upper limb arteries (343 cases), and branches of the aortic arch (69 cases), for a total of 1,896 cases. Thus, it was inferred that 27 cases had synchronous aneurysms in different sites. With regard to different arteries, ‘other’ in abdominal visceral arteries was most commonly affected (30.6%). Among these cases, internal iliac artery aneurysms accounted for a large portion, warranting a need for revision in the method of registration used. The next most commonly affected artery was the femoral artery (21.9%). In addition, 39.2% of cases were symptomatic, and the underlying cause was most commonly a degenerative disease (67.8%). Surgery included ligation and resection in 26.4%, replacement in 25.1%, coil embolization in 23.2%, and stent grafting in 16.3%, in line with 2013 data. The total number of surgical cases was 2,036, revealing that 8.2% of cases received multiple procedures, or different procedures were selected for the treatment of synchronous multiple aneurysms, as seen in 2013.

**Table:** Table 2-3 Peripheral artery aneurysm

Aneurysm	Cases	Gender		Mortality		Ruptured aneurysm			Etiology					Treatment procedure						Graft material for open surgery			
		Male	Female	30-day mortality	Hospital mortality	Cases	30-d mortality	Hospial mortality	Degenerative	Vasculatis*^9)^	Infected	Traumas	Others	Replacement	Exclusion with bypass	Ligation/resection	Stent graft	Coil embolization	Others	Polyester	ePTFE	Autogenous vessel	Others
Aortic arch branches																							
Carotid	8	6	2	0	0	0	0	0	4	0	1	0	3	2	1	2	3	2	1	1	1	1	0
Vertebral	0	0	0	0	0	0	0	0	0	0	0	0	0	0	0	0	0	0	0	0	0	0	0
Subclavian	47	35	12	1	0	0	0	0	31	3	1	5	7	9	10	5	11	12	6	9	8	1	0
Multiple in arch branches	0	0	0	0	0	0	0	0	0	0	0	0	0	0	0	0	0	0	0	0	0	0	0
Others	14	10	4	0	0	0	0	0	9	0	0	0	5	2	0	6	4	5	0	1	1	0	0
Upper limb artery																							
Axillar	19	12	7	0	0	0	0	0	18	0	0	0	1	14	4	1	0	0	1	1	10	7	0
Brachial	169	97	72	1	5	0	0	0	43	1	23	35	67	24	11	81	0	1	56	4	10	20	1
Forearm-hand	113	64	49	0	1	1	0	0	38	1	13	23	38	4	2	89	0	0	23	0	0	5	1
Others	42	23	19	0	0	0	0	0	17	0	7	2	16	3	1	28	0	1	11	1	3	1	0
Visceral artery																							
Celiac	15	12	3	0	0	0	0	0	13	0	0	0	2	2	0	3	4	8	3	0	0	2	1
Hepatic	24	18	6	0	0	3	0	0	15	0	4	1	4	5	5	6	0	8	2	0	3	4	0
Splenic	63	34	29	0	0	0	0	0	55	0	1	3	4	3	2	14	1	41	3	0	3	1	0
Superior mesenteric	21	20	1	0	1	1	0	0	14	1	2	0	4	2	2	3	6	7	1	0	0	4	0
Renal	72	40	32	0	1	0	0	0	64	0	2	0	6	13	2	23	11	22	14	1	1	9	0
Others	572	476	96	10	11	6	1	1	520	1	13	4	34	94	15	39	192	294	10	93	10	3	1
Lower limb artery																							
Femoral	410	330	80	8	13	1	0	0	199	5	55	45	106	166	25	142	14	10	78	81	82	28	2
Popliteal	214	163	51	3	2	0	0	0	192	3	2	4	13	116	73	31	1	0	15	20	59	111	0
Others	93	69	24	2	3	0	0	0	59	2	3	7	22	19	4	27	14	26	11	13	3	5	0
Total	1869	1383	486	25	37	12	1	1	1268	17	125	128	331	469	152	494	255	433	233	221	187	198	6

＊9) Including TAO, Takayasu aortitis, collagen disease related vasculitis, Behcet disease, fibromuscular dysplasia. Abbreviations; Y-graft: Y-shape artificial graft; T-graft: straight artificial graft; Polyester: polyester artificial graft such as Dacron graft; ePTFE: expanded polytetrafluoroethylene graft

## 2. Revascularization for Chronic Arterial Occlusion (Table 3)

### 1) Arteries of the arch branches, upper limbs, and abdominal viscera

In 2014, we observed an increase in the number of cases related to the carotid artery and others. Apart from little minor changes (observed in the vertebral artery, subclavian artery, multiple lesions of the aortic arch branches, and axillary artery-upper limb artery), we observed no major changes. With regard to the carotid artery, we noticed a remarkable increase in carotid endarterectomy (CEA), which was probably due to atherosclerosis, and the increase in bypass surgery thought to evolve to debranching surgery. In 42% of overall cases, revascularization was performed using debranching surgery associated with TEVAR/EVAR, revealing its rising tendency since 2013. Thus, it is inferred that stent grafting was performed for anatomically complex cases of aortic aneurysm.

**Table:** Table 3 Reconstruction for chronic arterial occlusive diseases*^10)^ Table 3-1 Arterial reconstruction for aortic arches

Aortic branches	Cases	Gender		Mortality	Background	Etiology						Revascularization procedures												Graft materials*^14)^				Previous reconstruction					Revision site				
		Male	Female	30-day mortality	Dialysis	ASO	TAO	Vasculitis*^11)^	Takayasu arteritis	Debranch for EVAR/ TEVAR	Others	CAS		CEA		PTA/stent*^13)^	Replacement	Visceral artery bypass	Internal iliac artery bypass	Anatomical bypass	Carotid-subclavian bypass	Axillo-axillar bypass	Others	Polyester	ePTFE	Autogenous veins	Others	None	Once	Twice	Three times and more	Unclear	Host artery stenosis/occlusion	Graft/EVT stenosis	Graft/EVT occlusion	Other	Unclear
												Cases	Brain complication*^12)^	Cases	Brain complication*^12)^	Cases																					
Carotid artery	84	69	15	1	3	61	0	2	1	15	5	7	0	50	2	5	2	0	1	3	14	10	4	7	14	1	1	78	4	0	1	1	3	0	0	2	0
Vertebral artery	2	0	2	0	0	1	0	0	0	1	0	0	0	0	0	1	0	0	0	0	1	0	0	0	1	0	0	2	0	0	0	0	0	0	0	0	0
Subclavian artery	116	86	30	1	5	78	2	0	2	26	8	1	0	0	0	50	1	0	0	5	21	43	8	19	44	1	0	106	5	1	2	2	5	1	1	1	0
Multiple lesions of arch branches	7	4	3	1	0	4	0	0	2	1	0	0	0	0	0	2	0	0	0	1	0	4	1	2	3	0	0	6	0	0	0	1	0	0	0	0	0
Upper limb including axillar artery	93	68	25	3	43	67	1	0	0	6	19	0	0	1	0	41	2	0	0	10	2	9	33	10	8	7	0	69	15	5	4	0	11	2	5	6	0
Celiac/Superior mesenteric artery	73	49	24	2	11	54	0	0	2	5	12	0	0	0	0	30	4	19	4	3	0	0	15	7	1	0	0	64	8	1	0	0	7	0	1	1	0
Renal artery	88	65	23	1	1	73	0	0	0	4	11	0	0	0	0	76	2	5	0	2	0	0	3	3	3	1	0	80	6	2	0	0	5	2	1	0	0
Others	321	254	67	7	12	26	0	0	0	281	14	0	0	0	0	25	0	24	9	32	118	155	46	124	123	2	8	306	10	3	0	2	10	1	0	2	0
Total	754	576	178	16	73	357	3	2	4	320	68	8	0	51	2	224	11	46	13	51	141	210	106	164	183	12	8	683	47	11	7	6	39	6	8	12	0

＊10) Bypass surgery combined with endovascular treatment is counted in both bypass category (**Table 3-2**) and endovascular category (**Table 3-5**). ＊11) Including TAO, Takayasu arteritis, coarctation of aorta, collagen disease related vasculitis, Behcet disease, fibromuscular dysplasia. ＊12) Postoperative irreversible brain complication. ＊13) Including percutaneous transluminal angioplasty (PTA), stent, and other endovascular means such as catheter atherectomy. ＊14) Only for open surgery.

**Table:** Table 3-2 Arterial reconstruction for chronic lower limb ischemia

From aorta to lower limb arterial systems	Cases	Gender		Mortality	Dialysis cases	Etiology						Graft materials				Previous reconstruction					Revision site				
		Male	Female	30-day mortality		ASO	TAO	Vasculitis	Takayasu arteritis	Debranch for TEVAR/EVAR	Others	Polyester	ePTFE	Autogenous veins	Others	None	Once	Twice	Three times and more	Unclear	Host artery stenosis/occlusion	Graft/EVT stenosis	Graft/EVT occlusion	Other	Unclear
Aorto-aortic bypass	57	48	9	0	3	53	0	0	1	0	3	40	17	1	0	47	6	4	0	0	7	1	2	0	0
Infrarenal aortic reconstruction (suprarenal clamp)	44	36	8	0	1	37	1	2	0	0	4	41	2	0	1	41	2	1	0	0	2	1	0	0	0
Aorto-femoral bypass*^15)^	632	505	127	9	49	603	4	4	2	4	15	458	182	38	10	542	61	12	12	5	49	7	23	5	1
Femoro-popliteal (above the knee) bypass	1,859	1,387	472	17	272	1,847	6	2	0	0	4	350	1,260	349	36	1,384	335	77	57	6	304	31	91	38	5
Infrapopliteal arterial bypass	1,879	1,390	489	26	621	1,817	23	12	0	0	27	84	414	1,397	95	1,254	386	113	113	13	381	45	139	31	16
Femoro-popliteal (below the knee) bypass	699	531	168	3	181	681	6	3	0	0	9	38	277	382	36	457	153	45	36	8	148	14	61	9	2
Femoro-crural/pedal bypass*^16)^	1,210	883	327	23	453	1,166	17	9	0	0	18	47	156	1,045	60	815	241	70	79	5	241	33	80	22	14
Others	179	141	38	2	44	167	2	0	0	1	9	46	90	44	3	122	38	10	9	0	36	11	7	2	1
Total	4,434	3,340	1,094	52	949	4,313	33	19	3	5	61	934	1,828	1,734	138	3,227	785	214	184	24	744	94	252	70	23

＊15) Including aorto-iliac bypass or ilio-femoral bypass. ＊16) Including popliteal-crural (or pedal) bypass.

**Table:** Table 3-3 Extra-anatomical bypass*^17)^

Extra-anatomical bypass	Cases	Gender		Mortality	Dialysis cases	Etiology				Graft materials				Previous reconstruction					Revision site				
		Male	Female	30-day mortality		ASO	TAO	Debranch for TEVAR/EVAR	Others	Polyester	ePTFE	Autogenous veins	Others	None	Once	Twice	Three times and more	Unclear	Host artery stenosis/occlusion	Graft/EVT stenosis	Graft/EVT occlusion	Other	Unclear
Carotid-subclavian bypass	141	112	29	4	1	7	1	131	2	74	72	1	5	138	3	0	0	0	2	0	0	1	0
Axillo-axillar bypass	216	165	51	8	7	32	0	175	9	86	133	1	7	205	9	1	1	0	7	1	1	2	0
Axillo-femoral bypass*^18)^	345	257	88	6	34	328	2	0	15	115	227	16	7	284	39	15	6	1	27	5	18	9	1
Femoro-femoral crossover bypass	890	730	160	9	64	855	0	10	25	269	585	60	8	701	136	22	28	3	123	10	39	14	0
Others	111	88	23	2	17	105	1	1	4	28	63	12	6	74	17	7	12	1	16	7	10	2	1
Total	1,609	1,276	333	25	118	1,303	4	247	55	531	1,027	85	29	1,315	198	45	46	5	172	22	67	26	2

＊17) Cases underwent extraanatomical bypass because of graft infection should not be included this category. Those cases are listed in vascular complication (**Table 6**). ＊18) A case underwent axillo-femoro-femoral crossover bypass is counted as one case. A case combined with additional contralateral side of axillo-femoral bypass as second staged surgery is counted as 2 cases.

**Table:** Table 3-4 Thromboendarterectomy*^19)^ for chronic lower limb ischemia

Thromboendarterectomy	Cases	Gender		Mortality	Dialysis cases	Etiology				Previous reconstruction					Revision site				
		Male	Female	30-day mortality		ASO	TAO	Debranch for TEVAR/EVAR	Others	None	Once	Twice	Three times and more	Unclear	Host artery stenosis/occlusion	Graft/EVT stenosis	Graft/EVT occlusion	Other	Unclear
Aorto-iliac lesion	84	65	19	1	12	82	0	1	1	68	9	1	4	2	10	1	3	0	0
Femoro-popliteal lesion	1,039	791	248	4	247	1,028	0	0	11	809	142	43	34	10	164	19	21	15	0
Others	121	93	28	0	24	114	1	0	6	94	15	4	5	3	14	2	5	3	0
Total	1,223	932	291	5	278	1,204	1	1	17	956	162	47	43	14	184	22	28	18	0

＊19) Including patch plasty.

**Table:** Table 3-5 Endovascular treatment for chronic lower limb ischemia*^13)^

Endovascular treatment	Cases	Gender		Mortality		Dialysis cases	Etiology				Previous reconstruction					Revision site				
		Male	Female	30-day mortality	Hospital mortality		ASO	TAO	Debranch for TEVAR/EVAR	Others	None	Once	Twice	Three times and more	Unclear	Host artery stenosis/occlusion	Graft/EVT stenosis	Graft/EVT occlusion	Other	Unclear
Aorto-iliac lesion*^20)^	3,113	2,536	577	17	34	365	3,073	4	4	32	2,547	360	93	86	27	365	72	56	30	16
Femoro-popliteal lesion*^20)^	2,704	1,895	809	25	54	772	2,691	4	0	9	1,773	495	192	218	26	608	155	95	41	6
Infrapopliteal-ankle lesion*^20)^	1,283	843	440	26	52	602	1,266	4	0	13	722	238	121	177	25	343	96	70	24	3
Others	126	76	50	2	8	77	124	0	0	2	33	20	18	49	6	43	17	21	6	0
Total (number of regions underwent EVT)*^20)^	6,324	4,734	1,590	57	117	1,494	6,255	12	4	53	4,514	964	349	422	75	1,136	285	202	93	19
Total (number of limbs underwent EVT)*^21)^	5,481	4,150	1,331	45	90	1,201	5,415	12	4	50	3,986	821	282	326	66	932	233	166	85	13

＊20) When endovascular treatment performed for multiple regions, the case should be counted in each regions (If a case underwent endovascular treatment in both aorto-iliac and femoro-popliteal region, this case can be counted one in aorto-iliac, and one in femoro-popliteal region). ＊21) Counting the patients number not treated regions. When a case underwent endovascular treatment in multiple region, the case is counted as one case. Abbreviations; ASO: arteriosclerosis obliterans; TAO: thromboangiitis obliterans (Buerger’s disease); CAS: carotid artery stenting; CEA: carotid endarterectomy; PTA: percutaneous transluminal angioplasty; EVT: endovascular treatment; IIA: internal iliac artery

### 2) Anatomical bypass, extra-anatomical bypass, and endovascular treatment for the aorta to arteries of the lower limb region

**Figure 4:**
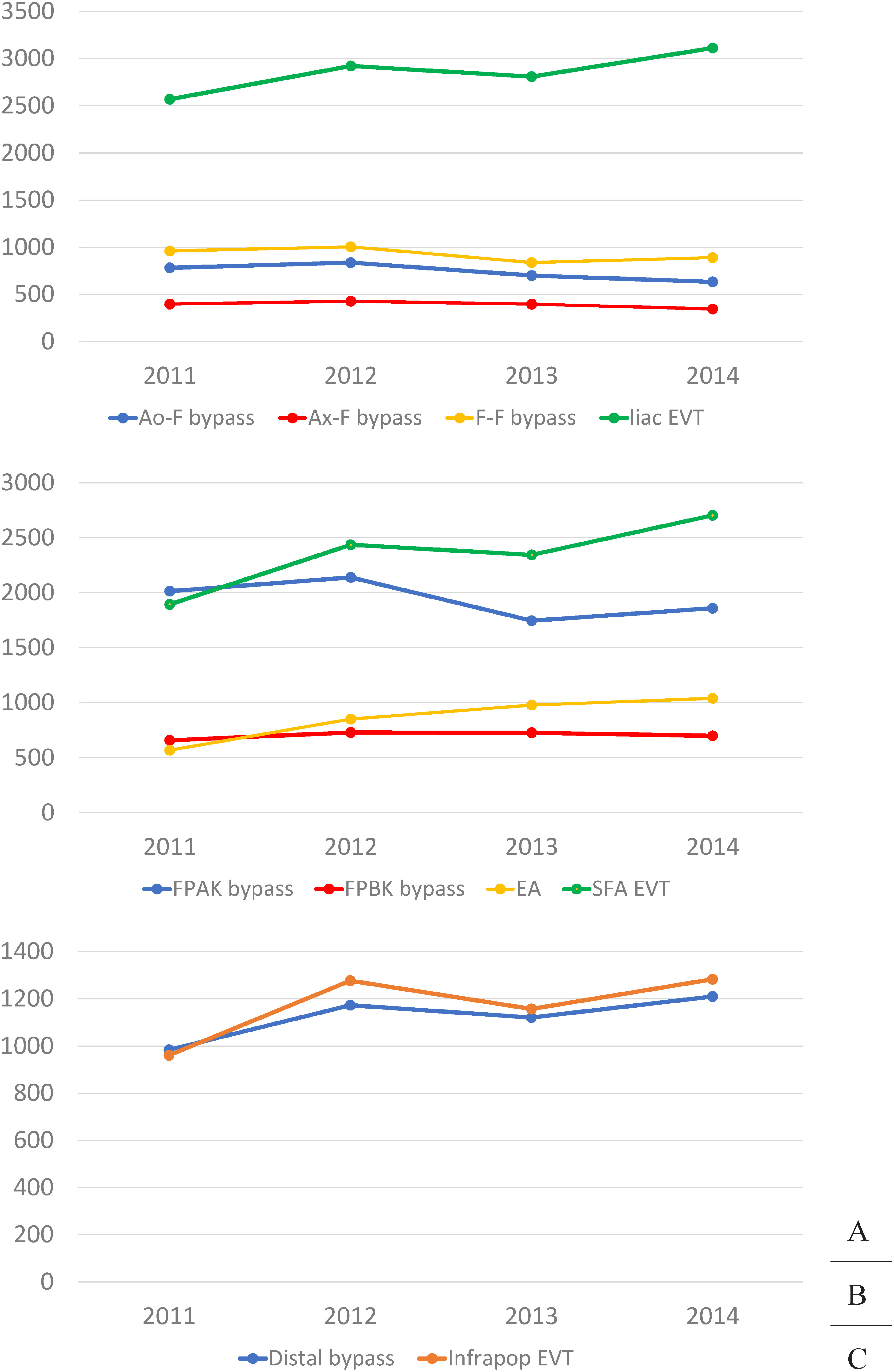
Fig. 4 The annual trends of the number of arterial reconstructions in aorto-iliac (**A**), femoro-popliteal (**B**), and crural/pedal region (**C**), comparing open repair and endovascular treatment.

Ao-F: aorto-femoral; Ax-F: axillo-femoral; F-F: femoro-femoral crossover; EVT: endovascular treatment; FPAK: femoro-popliteal (above the knee); FPBK: femoro-popliteal (below the knee); SFA: superﬁcial femoral artery; EA: endarterectomy

**Aortic–iliac artery region**: Anatomic bypass surgery for lesions of the aorto-iliac artery region showed very little changes (from 700 cases in 2013 to 733 cases in 2014), and there was no observed change in the vascular graft used. With regard to extra-anatomical revascularization (such as axillo-femoral bypass, and femoro-femoral bypass), we observed a decrease in the former and an increase in the latter as indicated from 396 and 838 in 2012 to 345 and 890 in 2014. However, there was no significant change observed in the total number of cases. The proportion of cases with a history of previous revascularization was higher; 20% for extra-anatomical bypass, compared to 13% for anatomical bypass (**Fig. 4A**).

**Superficial femoral artery region**: we observed an increase in the number of cases treated by femoral above-knee popliteal artery bypass (1,746 cases in 2013 to 1,859 cases in 2014), less than the number of endovascular interventions for the same site. Furthermore, we observed a history of previous revascularization in 25%, with an autogenous vein used for the graft in 19% of these cases (**Fig. 4B**).

**Infrapopliteal artery revascularization**: In 2013, femoral below-knee popliteal artery bypass and femoro-crural/pedal artery bypass were performed in 726, and 1,121 cases, respectively. Meanwhile, in 2014, the corresponding data indicated 699 and 1,210 cases, respectively, with an increasing tendency of crural/pedal artery bypass observed. Dialysis patients accounted for 37% of crural/pedal artery bypass cases, suggesting an increase of such cases in more severe patients. Moreover, 33% of cases had a history of previous revascularization, with autogenous vein used as the graft in 76% (**Fig. 4C**).

**Thromboendarterectomy**: With regard to thromboendarterectomy of the arteries in the lower extremities in the femoro-popliteal region, there were 978 cases in 2013. However, it increased to 1,039 cases in 2014, of which 24% were dialysis patients. It was thought that access to endovascular treatment was difficult, and that there was an increase in angioplasty for lesions of the common femoral artery. (**Fig. 4B**).

**Endovascular treatment**: The total number of endovascular treatment cases increased by 13% to approximately 1,000 cases in 2013. Among these, 25% were dialysis patients, indicating an increase in the proportion of dialysis patients. There was a slight increase of 3% in surgical revascularization (bypass and thromboendarterectomy) from 6,758 in 2013 to 6,892 in 2014. Conversely, we observed a significant increase in endovascular treatment. Among which, we observed the greatest rate increase at 15% in the femoral-popliteal artery region (from 2,344 cases in 2013 to 2,704 cases in 2014). From 2012 to 2013, in the femoral artery region, we noticed the data was affected by the fact that health insurance started the reimbursement for various nitinol stents. The rate of increase was approximately 10% for both the iliac artery and lower extremity regions. (**Figs. 4A**–**4C**).

We compared results with data of the Japanese registry of endovascular treatment (J-EVT), with University Hospital Medical Information Network (UMIN) as the parent body of UMIN, that was published on the homepage of the Japanese Association of Cardiovascular Intervention and Therapeutics (CVIT). In 2014, endovascular treatment was performed in 5,851 cases in the aortoiliac region by departments of cardiovascular medicine,^2)^ and there were a total of 4,980 cases of anatomical and non-anatomical reconstruction, and endovascular treatment conducted by a vascular surgeon, accounting for 46.0% of cases overall, indicating no change from 47.0% in 2012. In the J-EVT for 2014, in the superficial femoral artery region, endovascular treatment was performed in 7,592 cases,^2)^ and there was a total of 4,563 cases of femoral above-knee artery bypass performed by a vascular surgeon, accounting for 37.5% overall. This indicated an increase of 3.7% from 2012. In the J-EVT, in the below-knee artery region, endovascular treatment was performed in 4,187 cases in 2014,^2)^ and there were a total of 3,192 cases including femoral below-knee popliteal bypass, femoral below-knee/pedal artery bypass, and endovascular treatment performed by a vascular surgeon, accounting for 43.3% overall, indicating a reduction of 3.9% from 2012.

## 3. Revascularization for Acute Arterial Occlusion (Table 4)

We recorded 4,799 cases of acute arterial occlusion excluding vascular trauma. Among these, approximately 80% occurred in peripheral vessels of the abdominal aorta, of which thrombosis and embolism accounted for approximately half, similar to the previous year. In addition, we recorded 5,527 cases of occlusion in different regions, and thus it was inferred that occlusion occurred in multiple sites in 728 cases (13%), similar to the previous year. Thrombolytic therapy, which was included as an item since 2013, was performed in 70 cases (1.5%). Overall, the proportion of percutaneous transluminal angioplasty (PTA; with or without stenting) was 12.6%, indicating a slight increase from 10.8% in the previous year. In the femoral popliteal artery region, it was difficult to calculate the precise proportion for each treatment method as several treatment combinations were included; such as thrombectomy with bypass surgery, and thrombectomy with endovascular treatment. However, there were 337 cases of bypass surgery and 337 cases of endovascular treatment (PTA with or without stenting, and thrombolysis). This reveals that endovascular treatment was performed for 50% of cases in this region. A synthetic graft was used for bypass surgery in 67.6% (71.6% the previous year) in the femoro-popliteal region, and 54.8% in infrapopliteal arteries (50.0% the previous year). The rate of synthetic graft usage in infrapopliteal artery bypass for acute aortic occlusion was extremely high compared with 15.5% of infrapopliteal artery bypass for chronic arterial occlusion. The operative mortality was 11.0% in the abdominal aorta-iliac artery region, 8.1% in the femoro-popliteal artery, 9.3% in the crural artery, and 5.1% in the pedal artery, indicating clearly worse prognosis compared to normal elective vascular surgery. There were 105 cases of acute occlusion of celiac artery/superior mesenteric artery (2.2%). Of these, the operative mortality was 21.0%, and in-hospital mortality was 25.7%, which was as per previous years, indicating an extremely poor prognosis.

**Table:** Table 4 Revascularization for acute arterial occlusive disease*^22)^

Obstructive artery*^23)^	Cases	Gender		Mortality		Etiology			Procedure						Graft materials for open surgery			
		Male	Female	30-day mortality	Hospital mortality	Embolism	Thrombosis*^24)^	Others	Thrombectomy±patch*^25)^	Bypass	Replacement	PTA±stent	Thrombolysis	Other	Autogenous vessel	Polyester	ePTFE	Others
Carotid artery	21	13	8	0	0	3	6	12	8	7	2	1	0	4	4	3	2	0
Subclavian artery	61	31	30	2	4	29	16	16	35	16	2	7	0	2	2	9	8	2
Axillar artery	75	26	49	3	4	39	33	3	63	6	0	7	0	1	0	2	6	0
Brachial artery	752	383	369	32	44	356	381	15	648	13	4	36	3	75	8	19	13	2
Celiac/superior mesenteric artery	105	64	41	22	27	48	26	31	51	24	0	15	5	14	19	4	3	0
Renal artery	17	12	5	3	4	5	2	10	0	4	0	11	0	2	1	3	1	0
Abdominal aorta-iliac artery	806	573	233	89	114	303	382	121	498	235	23	178	10	32	20	132	140	8
Femoro-popliteal artery	2,582	1,625	957	210	259	1,101	1,350	131	2,096	337	31	297	40	122	155	138	214	14
Crural artery	837	534	303	78	97	368	435	34	673	77	5	132	23	48	53	42	26	3
Pedal artery*^26)^	39	29	10	2	6	17	20	2	24	7	2	2	0	6	5	4	0	0
Others	232	142	90	10	12	38	164	30	168	20	5	44	7	16	8	24	15	1
Total	4,799	2,986	1,813	363	457	1,973	2,450	376	3,651	658	62	603	70	291	237	337	376	27

＊22) Cases with non-traumatic acute arterial occlusion are listed in this table. Please see **Table 5-1** for acute arterial occlusion by trauma. ＊23) The most proximal occluded artery name is described in case whose primary occluded artery could not be identified. ＊24) Cases with acute worsening occlusion of chronic arterial occlusive disease are excluded. Treatment for those cases are listed in **Table 3**. ＊25) If either thrombectomy or patch plasty is performed, cases are listed in this section. ＊26) Including acute occlusion of dorsalis pedis or planter artery.

## 4. Treatment for Vascular Trauma (Table 5)

The sites, causes, surgical procedure, and type of graft used in vascular trauma in the NCD-registered data of 2014 are presented in **Table 5**. We recorded 2,088 cases of artery and venous traumas. The most common cause of vascular trauma was iatrogenic at 1,435 cases (69%, with traffic accident-related injuries in 141 cases (7%), and work-related injuries in 156 cases (7%). The most common site was the lower limb arteries (46%), followed by the upper limb arteries (17%), and the abdominal aorto-iliac artery (9%). The surgical treatment method was registered in 2,182 cases, and according to surgical procedure, direct closure accounted for 56% of overall cases. Vascular grafts were used in 270 cases, and autologous vessels were used in approximately 49% of these cases.

**Table:** Table 5 Treatment for vascular trauma Table 5-1 Arterial trauma*^27)^

Injured artery	Cases	Gender		Mortality		Cause of trauma				Procedure							Status of injured artery*^28)^						Prosthesis			
		Male	Female	30-day mortality	Hospital mortality	Traffic accident	Labor accident	Iatrogenic	Others	Direct closure	Patch plasty	Replacement	Bypass	Endo-vascular	Ligation	Others	Obstruction/stenosis*^28)^	bleeding without specification*^29)^	GI fistula	Non-GI fistula	Pseudo-aneurysm	Others	Autogenous vessel	Polyester	ePTFE	Others
Carotid artery	31	23	8	4	5	2	0	21	8	13	0	2	3	7	6	3	2	21	2	2	1	3	1	1	3	1
Subclavian artery	40	22	18	6	7	3	1	27	9	19	2	0	3	9	4	4	1	18	0	1	8	12	2	2	1	1
Axillar artery	14	9	5	0	0	2	4	4	4	3	0	1	6	1	2	2	6	5	0	0	5	1	4	0	3	0
Brachial artery	303	179	124	6	8	9	16	255	23	224	10	4	15	6	32	29	28	57	0	6	180	44	25	1	2	0
Descending aorta (thoracic/thoracoabdominal)	41	27	14	4	6	14	5	14	8	8	1	2	0	17	1	12	0	28	2	2	7	5	0	1	1	2
Celiac/superior mesenteric artery	41	29	12	7	9	7	3	16	15	11	0	1	2	18	3	6	6	26	4	3	2	3	3	1	0	0
Renal artery	16	14	2	3	3	3	1	10	2	2	0	0	0	6	4	4	1	11	0	3	0	1	0	0	0	0
Abdominal aorta-iliac artery	188	110	78	26	34	17	10	115	46	47	2	24	26	65	23	17	30	97	6	7	18	36	2	28	21	1
Femoro-popliteal artery	924	641	283	119	153	27	50	720	127	681	29	40	68	28	64	53	88	252	1	15	302	290	78	20	36	2
Crural artery	43	29	14	0	0	5	10	21	7	17	2	1	8	5	10	4	6	17	0	0	11	9	8	1	0	0
Others	325	214	111	18	29	50	44	149	82	102	2	5	12	77	79	58	34	176	5	9	53	51	8	2	7	1
Total	1,946	1,285	661	191	252	137	143	1,339	327	1,120	48	79	138	231	224	191	196	699	20	48	583	451	130	53	73	7

＊27) Cases with vessel injury involving both vein and accompanying artery are listed in **Table 5-1**. ＊28) Iatrogenic pseudoaneurysm in endovascular treatment is listed in **Table 5-1**. ＊29) Including arterial dissection. ＊30) Without GI fistula or non-GI fistula. Abbreviation; GI: gastro-intestinal

**Table:** Table 5-2 Venous trauma*^28)^

Injured veins	Cases	Cause of trauma				Procedure							Prosthesis			
		Traffic accident	Labor accident	Iatrogenic	Other	Direct closure	Patch plasty	Replacement	Bypass	Endo-vascular	Ligation	Others	Autogenous vessel	Polyester	ePTFE	Others
Superior vena cava	6	1	0	5	0	4	0	1	0	1	0	0	0	0	1	0
Inferior vena cava	12	1	0	6	5	6	0	0	0	3	1	2	0	0	0	0
Brachiocephalic-subclavian vein	8	0	1	6	1	6	0	0	0	0	2	1	0	0	0	0
Iliac-femoral-popliteal vein	64	1	3	50	10	56	0	2	2	0	9	1	1	1	2	0
Others	55	1	9	29	16	29	0	1	1	0	19	8	1	0	1	0
Total	142	4	13	96	29	98	0	4	3	4	30	12	2	1	4	0

**Figure figure5:**
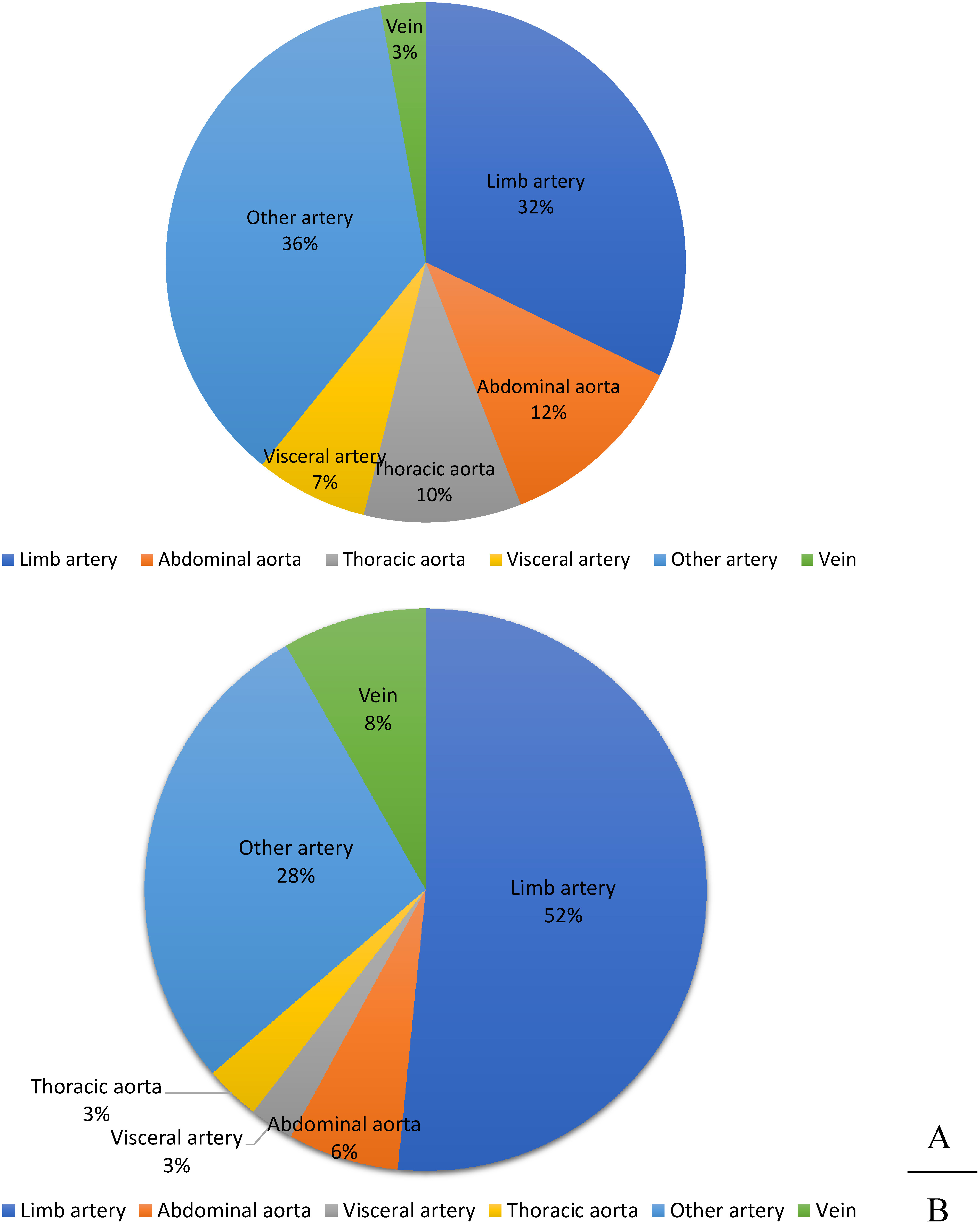
Fig. 5 Location of vascular injury in year 2014. Injured vessels by trafﬁc accident (**A**) and work-related accident (**B**).

### 1) Iatrogenic vascular trauma

From the 1,435 cases in 1,448 sites of iatrogenic trauma, the most common site affected was the lower limb arteries (approximately 51%), followed by the upper limb arteries (approximately 20%). Many of these cases appeared to be attributed to complications at puncture sites associated with endovascular catheter examinations and treatment procedures.

### 2) Traffic accidents (Fig. 5A)

Among 141 cases and 143 sites of traffic accident-related vascular trauma, the most common site affected was the upper and lower limb arteries, accounting for approximately 32%. This is thought to be attributed to the fact that the vessels of the limbs are near the body surface and are easily affected by direct external force. The second most common site was the abdominal aorto–iliac arteries (12%), followed by the descending aorta/thoracoabdominal aorta (10%), and visceral artery (7%).[Fig figure5]

### 3) Work-related (Fig. 5B)

There were 156 cases and 157 sites registered that were considered to be work-related injuries such as falls from heights and machinery-related injuries. We found out that injuries to the arteries of the extremities accounted for 52%. As noted above, these arteries are close to the body surface, making them prone to external impact.

In conclusion, we summarized the registration status of vascular injuries of 2013 in the NCD database. Compared to 2013, the total number of registered cases increased slightly. However, there was no significant difference in the cause of trauma, trauma site, type of graft used, and treatment procedure used.

## 5. Surgery for Vascular Complications after Revascularization (Table 6)

Very few cases registered involved the thoracic to thoracoabdominal artery regions, and therefore, the peripheral region of the extremities were of greater concern.

**Table:** Table 6 Revascularization for vascular complication after revascularization Table 6-1 Graft infection

Position of infected garft	Cases	Mortality		Status of infected graft				Procedure for graft infection			Material for revision or redo surgery				
		30-day mortality	Hospital mortality	Sepsis	Graft-GI fistula*^31)^	Graft-skin fistula*^31)^	Others	In-situ replacement	Extra-anatomical bypass	Others	Polyester	ePTFE	Autogenous vessel	Cryo-preserved homograft	Others
Descending thoracic aorta	5	3	3	4	0	0	1	1	0	4	1	0	0	0	0
Thoracoabdominal aorta	5	0	0	3	0	1	1	1	0	3	2	2	0	0	0
Abdominal aorta-iliac artery	0	0	0	0	0	0	0	0	0	0	0	0	0	0	0
Abdominal aorta-femoral artery	0	0	0	0	0	0	0	0	0	0	0	0	0	0	0
Femoro-distal artery	0	0	0	0	0	0	0	0	0	0	0	0	0	0	0
Others*^32)^	254	17	31	60	3	110	100	15	0	202	17	64	26	0	5
Total	264	20	34	67	3	111	102	17	0	209	20	66	26	0	5

＊31) Including anastomotic disruption. ＊32) Cases with graft infection involving aortic arch branch or upeer limb artery are listed on this column. Abbreviation; GI: gastrointestinal

**Table:** Table 6-2 Anastomotic aneurysm*^33)^

Location of anastomotic aneurysm	Cases	Mortality	Cause of aneurysm treated at the primary operation					Repair procedure				Material for repair surgery			
		30-day mortality	Degenerative	Takayasu arteritis*^34)^	Other vasculitis*^35)^	Infection	Others	Replacement	Exclusion and bypass	Stent graft	Others	Polyester	ePTFE	Autogenous vessel	Others
Aortic arch branch	8	1	2	1	5	0	1	0	2	0	1	5	2	2	2
Upper limb artery including axillar artery	33	3	10	2	5	0	0	3	25	3	2	1	27	0	2
Thoracic aorta	10	0	0	0	8	0	0	0	2	1	0	5	4	4	0
Splanchnic artery	5	1	1	0	1	0	1	0	3	0	1	1	3	1	2
Renal artery	2	0	0	0	2	0	0	0	0	0	0	0	2	1	0
Abdominal aorta	29	1	9	1	23	0	0	1	5	10	1	15	4	17	2
Iliac artery	16	2	2	0	12	0	1	0	3	4	1	7	4	7	1
Femoral artery	47	1	9	0	30	0	0	4	13	15	6	0	28	12	10
Popliteal or more distal lower limb artery	10	1	5	0	6	0	0	1	3	2	1	1	7	0	1
Total	152	10	37	4	84	0	3	9	56	33	13	34	75	41	19

＊33) Cases with infected pseudoaneurysm located at the anastomotic site to the artificial graft are listed in **Table 6-1**. ＊34) Including the atherosclerotic aneurysm. ＊35) Including TAO, collagen disease, Behcet disease, and fibromuscular dysplasia.

**Table:** Table 6-3 Autogenous graft aneurysm

Revascularization area	Cases	Mortality	Repair procedure		
		30-day mortality	Replacement	Bypass	Others
Vesceral artery	1	0	0	0	1
Upper limb artery	23	0	4	3	16
Lower limb artery	22	1	4	9	10
Others	6	0	0	0	6
Total	52	1	8	12	33

**Table:** Table 6-4 Graft degeneration

Revascularization	Cases	Mortality	Initial revascularization procedure				Degenerative material			Repair procedure					Material for repair surgery		
		30-day mortality	Replacement	Bypass	Stent graft	Others	Polyester	ePTFE	Others	Replacement	Bypass	Stent graft	Patch plasty	Others	Polyester	ePTFE	Others
Descending thoracic aorta	1	0	0	0	1	0	0	0	1	0	0	1	0	0	0	0	1
Thoracoabdominal aorta	1	0	1	0	0	0	0	1	0	1	0	0	0	0	0	0	1
Abdominal aorta-femoral artery	16	0	10	4	2	0	13	0	3	8	1	6	0	1	9	3	2
Femoro-popliteal artery	14	0	3	10	0	1	10	3	1	4	4	1	0	5	5	5	0
Others	21	0	5	6	0	10	8	10	3	8	3	1	0	9	5	9	2
Total	52	0	19	19	3	11	30	14	8	21	7	9	0	15	18	17	6

**Table:** Table 6-5 Repair operation for graft stenosis or acute thrombosis*^36)^

Initial procedure	Cases	Mortality	Repair procedure						Material for repair surgery			
		30-day mortality	Patch±thrombectomy	Replacement	Bypass	PTA±stent	Thrombolysis	Others	Polyester	ePTFE	Autogenous vessel	Others
Reconstruction of aorta or its primary branches	179	8	31	17	48	85	0	17	52	31	5	17
Revascularization of upper limb	117	0	54	16	15	29	0	17	4	40	15	8
Revascularization of lower limb	788	5	241	45	188	374	6	58	58	115	212	21
Total	1,078	13	326	78	248	486	6	91	114	184	231	46

＊36) Including stenosis such as the anastomotic stenosis, graft stenosis or occlusion, and restenosis at the site of endarterectomy.

**Figure 6:**
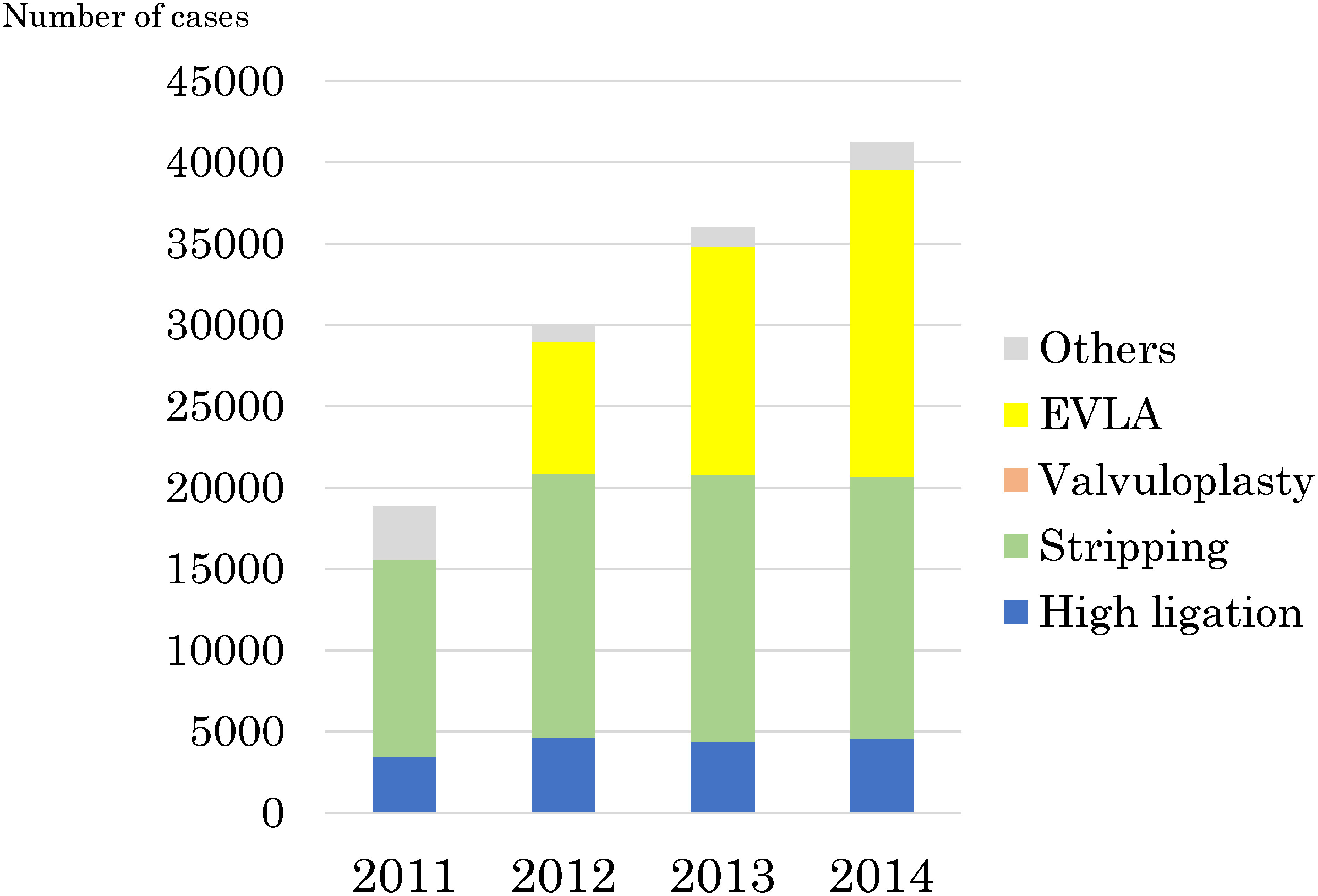
Fig. 6 Changes of varicose veins treatment in year 2011, 2012, 2013 and 2014.

EVLA: endovenous laser ablation

### 1) Vascular graft infection (Table 6-1)

We recorded 264 cases of vascular graft infection, which mostly occurred in the region classified as “other” (96.2%). It included the upper limb arteries, and there were no cases registered that involved the femoral artery-peripheral artery. Owing to the increase in cases of endovascular treatment for the lower limb arteries, it was expected that prosthetic graft revascularization would decrease. However, no cases were reported to use endovascular recanalization. The majority of other cases including those involving the upper limb arteries experienced prosthetic cutaneous fistula, of which, few were repaired by endovascular recanalization.

### 2) Anastomotic aneurysm (non-infectious) (Table 6-2)

Regional examination revealed that anastomotic aneurysms most commonly occurred in the femoral arteries (30.9%), followed by the axillary-upper limb arteries. The most frequent etiology was atherosclerosis in the lower limb arteries and abdominal aorta. However, in the upper limb arteries, “other” was the most common cause.

### 3) Autologous graft aneurysm (Table 6-3)

We did not observe a difference in the number of registered cases of autogenous graft aneurysms in the upper limb arteries, and lower limb arteries. Revascularization (replacement/bypass surgery) was performed in 59.0% of cases involving the lower limb arteries, and in 30.4% of those involving the upper limb arteries.

### 4) Vascular graft deterioration (Table 6-4)

We recorded 52 cases of vascular graft deterioration registered, of which the initial surgical procedure was replacement and bypass in 19 of them, and stent grafting in 3 cases. It is expected that recent changes in endovascular recanalization procedures will result in an increase in the number of cases of stent grafting.

### 5) Vascular graft stenosis and occlusion (Table 6-5)

According to records, cases of lower limb artery reconstruction were the most frequently registered (73.1%), and the underwent PTA±stenting (47.5%), followed by patch/thromboendarterectomy, and bypass surgery.

## 6. Venous Surgery (Table 7)

### 1) Varicose veins (Table 7-1)

We noticed a sharp increase in the number of surgeries, with 41,246 cases reported in 2014. This shows 2-fold increase compared to NCD-registered data of 2011. With regard to the surgical procedure, stripping (with or without sclerotherapy) was performed in 16,155 cases (39%), similar to the previous year. However, laser therapy (endovenous laser ablation (EVLA)) (with or without sclerotherapy) increased from 14,043 in 2013 to 18,861 cases in 2014 (46%), indicating that it was the most common surgical method (**Fig. 6**). With the coming of radio frequency (RF) in endovascular ablation from 2014, it may have been included in other procedures from then on. This basis demonstrates why endovascular ablation has gained popularity for the treatment of varicose veins.^2, 3)^

**Table:** Table 7 Venous surgery Table 7-1 Varicose veins

Varicose veins treatment	Cases*^37)^	Male	Female	30-day mortality
High ligation±sclerotherapy	4,533	1,484	3,048	0
Stripping±sclerotherapy	16,155	6,255	9,899	0
Valvuloplasty	1	1	0	0
Laser ablation±sclerotherapy	18,861	6,417	12,441	0
Others	1,696	448	1,284	0
Total	41,246	14,605	26,636	0

＊37) Only one procedure can be registered in one leg.

### 2) Deep vein thrombosis (including deep vein stenosis and occlusion) (Table 7-2)

We recorded 520 cases of surgery registered. The most indicated procedure was inferior vena cava filter placement in 299 cases (58%), followed by filter removal in 130 cases (25%), similar to the previous year. Catheter-directed thrombolysis (CDT) was performed in 31 patients (6%), while surgical procedures included thrombectomy in 67 cases (13%), bypass surgery (peripheral vein revascularization) in 3 cases (1%), and release of venous stenosis (by direct approach) in 13 cases (3%), indicating low incidences.

**Table:** Table 7-2 Deep vein thrombosis (including venous stenosis or obstruction)

Deep vein thrombosis treatment	Cases	Male	Female	30-day mortality
Thrombectomy	67	29	38	4
Catheter-directed thrombolysis*^38)^	31	16	15	0
Bypass (peripheral venous reconstruction)	3	0	3	0
IVC filter insertion*^39)^	299	121	178	8
IVC filter retrieval*^39)^	130	45	85	1
Direct surgery of stenosis*^40)^	13	6	7	0
Endoluminal treatment of stenosis*^40)^	18	4	14	1
Others	6	2	4	0
Total	520	207	313	10

＊38) Including the catheter-directed thrombolysis using hydrodynamic thrombectomy catheter. ＊39) including temporary IVC filter. ＊40) including obstructive lesions.

### 3) Upper limb and cervical vein stenosis and occlusion (Table 7-3)

We recorded 132 cases of surgery, fewer compared to that recorded in 2012. The most commonly registered procedure performed by endovascular treatment was repair of venous stenosis in 80 cases (61%).

**Table:** Table 7-3 Upper limb vein stenosis or obstruction

Treatment of vein stenosis (obstruction)	Cases	Male	Female	30-day mortality
Thrombectomy	24	11	13	1
Catheter-directed thrombolysis*^41)^	3	3	0	0
Bypass	9	6	3	1
SVC filter insertion*^42)^	0	0	0	0
Direct surgery of stenosis*^43)^	8	4	4	0
Endoluminal treatment of stenosis*^43)^	80	51	29	0
Others	12	5	7	0
Total	132	78	54	1

＊41) Including the catheter-directed thrombolysis using hydrodynamic thrombectomy catheter. ＊42) Including temporary IVC filter. ＊43) Including obstruction.

### 4) Vena cava revascularization (Table 7-4)

Here, we recorded 66 cases of related surgery; including inferior vena cava/primary branch reconstruction in 51 cases (77%), and superior vena cava/primary branch reconstruction in 15 cases (23%), a ratio of 3 : 1. The most common cause was tumors in 51 cases (77%), with an operative mortality in 5 cases (10%), and in-hospital mortality in 7 cases (14%), indicating poor outcomes. The surgical procedure involved replacement in 12 cases, patching in 10 cases, and bypass in 4 patients, with ePTFE the most frequently used method.

**Table:** Table 7-4 Vena cava reconstruction

Vena cava reconstruction	Cases	Mortality		Etiology			Treatment procedures					Material for open surgery			
		30-day mortality	Hospital mortality	Tumor	Thrombus	Others	Patch plasty	Bypass	Replacement	PTA±stent	Others	Autogenous vessel	Polyester	ePTFE	Others
SVC reconstruction	15	2	3	7	2	6	2	1	3	3	6	0	1	5	2
IVC reconstruction	51	3	4	44	3	4	8	3	9	4	27	6	1	9	7
Total	66	5	7	51	5	10	10	4	12	7	33	6	2	14	9

Abbreviations; SVC: superior vena cava; IVC: inferior vena cava

### 5) Budd-Chiari syndrome (Table 7-5)

Very few cases of surgery were registered in the database (7 cases), including percutaneous shunt creation in 6 cases, and open repair in only 1 case.

**Table:** Table 7-5 Budd-Chiari syndrome

Treatment 1	Cases	Gender		Mortality		Material for open surgery			
		Male	Female	30-day mortality	Hospital mortality	Polyester	ePTFE	Autogenous vessel	Others
Shunting	0	0	0	0	0	0	0	0	0
Percutaneous shunting	6	4	2	0	0	0	0	0	5
Surgical recanalization	1	0	1	0	0	0	0	0	0
Total	7	4	3	0	0	0	0	0	5

### 6) Other (Table 7-6)

We recorded 25 cases of plication and suture for venous aneurysms of deep veins, less compared to data in 2013. There was a rare case of surgery for venous aneurysm of the visceral vein.

**Table:** Table 7-6 Other surgery

Treatment	Cases	Gender		Mortality		Material for open surgery			
		Male	Female	30-day mortality	Hospital mortality	Polyester	ePTFE	Autogenous vessel	Others
Plication of deep venous aneurysm*^44)^	25	15	10	0	0	0	0	0	0
Plication of abdominal venous aneurysm*^45)^	1	1	0	0	0	0	0	0	0
Others	867	467	400	19	55	0	0	1	0
Total	893	483	410	19	55	0	0	1	0

＊44) Including patch plasty. ＊45) Including cases with access repair using artificial graft.

## 7. Other Vascular Diseases and Related Surgeries (Table 8)

Compared to 2012, we observed a decrease in the number of cases in 2013. However, this declining tendency disappeared in 2014, and the number of cases of vascular access surgery and lower limb amputation rather increased.

### 1) Popliteal artery entrapment syndrome (Table 8-1) and cystic adventitial disease (Table 8-2)

The number of cases greatly decreased in 2013 compared to 2012. However, in 2014, the number of cases showed very little change from 2013. These conditions are essentially rare, and further data is awaited to predict future trends.

**Table:** Table 8 Other vascular diseases Table 8-1 Popliteal artery entrapment syndrome

Treatment	Cases	30-day mortality
Myotomy	7	0
Revascularization	24	0
Total	28	0

**Table:** Table 8-2 Adventitial cystic disease

Treatment	Cases	30-day mortality
Cyst excision±patch plasty	27	0
Replacement	10	0
Bypass	4	0
Total	36	0

### 2) Thoracic outlet syndrome (Table 8-3)

In 2014, only 6 cases were treated. Apart from bypass surgery, the procedures were commonly performed by the orthopedic surgery department. Thus, we infer that this data did not reflect the actual number of cases.

**Table:** Table 8-3 Throracic outlet syndrome (TOS)

Treatment	Cases	Male	Female	30-day mortality	Type of TOS*^46)^		
					Neurogenic	Venous	Arterial
Rib resection*^47)^	2	2	0	0	0	1	1
Rib resection+scalenectomy	0	0	0	0	0	0	0
Bypass	4	2	2	0	0	0	4
Total	6	4	2	0	0	0	5

＊46) In the case with mixture type, the type having the most significant impact on the clinical symptom is listed. But, if the impacts are similar, multiple response is allowed. ＊47) Including cervical rib.

### 3) Vascular access surgery (Table 8-4)

The number of registered cases increased by 2,000 cases from the previous year, with an overall increase in procedures; including that for new access creation, repair, PTA, and shunt aneurysm repair. In the future, we predict an increase in the number of cases with the increase in artificial dialysis.

**Table:** Table 8-4 Vascular access operation

Treatment	Cases	30-day mortality
Arteriovenous access creation by autogenous material	12,549	134
Arteriovenous access creation by artificial material*^47)^	2,710	56
Open surgery for access repair	2,229	38
Endovascular access repair	6,688	31
Arterial transposition	415	18
Arteriovenous access aneurysm repair	433	4
Total	25,024	281

### 4) Surgical treatment for lymphedema (Table 8-5)

We recorded 53 cases in 2014, which was approximately half the number of cases in 2013. However, the actual number of cases remained unclear.

**Table:** Table 8-5 Surgery for lymphedema

Treatment	Cases	Male	Female	30-day mortality
Lymphovenous anastomosis	0	0	0	0
Lymph drainage operation	5	4	1	0
Resection	48	29	19	1
Total	53	33	64	1

### 5) Sympathectomy (Table 8-6)

This year, we recorded only 27 cases of sympathectomy, similar to the previous year, previewing a decrease in the future, though the indications of this procedure are fairly limited.

**Table:** Table 8-6 Sympathectomy

Sympathectomy	Cases	30-day mortality
Thoracic sympathectomy	14	0
Lumbar sympathectomy	13	0
Total	27	0

### 6) Upper limb and lower limb amputation (Tables 8-7 and 8-8)

While the number of cases of upper limb amputation remained unchanged, the number of lower limb amputations increased in contrast to the significant decrease recorded in the previous year. However, most procedures were performed by the department of orthopedic surgery. Therefore, to improve the treatment outcomes for severe lower limb ischemia in future, we have to consider data redistribution and tabulation of results across medical departments.

**Table:** Table 8-7 Amputation of upper limb

Amputation level	Cases	30-day mortality
Digit	20	0
Forearm/upper arm	2	0
Total	22	0

**Table:** Table 8-8 Amputation of lower limb*^48)^

Amputation level	Cases	30-day mortality	Etiology			
			ASO	DM-ASO	TAO	Others
Toe	519	12	205	273	4	37
Transmetatarsal	234	4	68	144	1	21
Lisfranc/chopart	32	4	13	13	5	1
Syme	3	0	0	3	0	0
Below-knee	232	9	81	138	2	11
Through-knee/above-knee	299	23	144	121	0	34
Hip	3	1	2	1	0	1
Total	1,322	53	513	693	12	104

＊48) Amputations not due to ischemia are not included. Abbreviations; ASO: arteriosclerosis obriterance; DM-ASO: diabetic ASO; TAO: thromboangiitis obliterans (Buerger’s disease)

## Conclusion

Following on from 2013, 2012, and 2011, when registration in the NCD began, we clarified an overall view of vascular surgery in 2014. Although only simple calculations, these data provide a glimpse of the current state of vascular surgery in Japan along with an understanding of the changes over time in the details of vascular surgery.

One of the main aims of participating in the NCD is to improve the quality of medical care using NCD data. Since data entry occurs simultaneously busy medical practice, limiting the entries to essential input parameters is a noble task to be addressed. However, to improve evaluation of the quality of medical care, the number of input items has increased each year from 2012 through 2014. Due to a fortunately low operative mortality for most cases of vascular surgery (except for major aortic vascular surgery), it should not be used as an indicator in evaluations. Thus, a future objective is to establish a function in the NCD with which risk adjusted quality of vascular surgical treatment at each institution can be compared with national standards. In 2018, the JSVS commenced a multicenter observational study of the selection of treatment by open surgery and stent grafting for ruptured abdominal aortic aneurysms. They underwent a retrospective study on treatment and prognosis of infected aneurysms of the abdominal aorta and common iliac artery as a model study. In 2019, they commenced a retrospective study investigating surgical procedures and prognosis for popliteal artery entrapment syndrome, and activities are ongoing to achieve these tasks. As from 2018, the JSVS started a public appeal for proposals of new research topics in the field of vascular surgery using NCD data in 2019. Furthermore, in order to improve data reliability, on-site visitations have commenced. In future, we hope to continue the development of a new vascular surgery database on the NCD together with all members of the JSVS. We sincerely hope that this database will serve to help provide high-quality medical care to patients with vascular disease.

## Appendix

Team responsible for analyzing the 2014 annual report as follows;

Database Management Committee of the Japanese Society for Vascular Surgery: Nobuya Zempo (Chairman), Nobuyoshi Azuma (Vice-chairman), Yukio Obitsu (Vice-chairman), Yoshinori Inoue, Hitoshi Okazaki, Hideaki Obara, Hirono Satokawa, Kunihiro Shigematsu, Ikuo Sugimoto, Hiroshi Banno, Naoki Fujimura, Akihiro Hosaka, Shinsuke Mii, Noriyasu Morikage, Terutoshi Yamaoka, Tetsuro Miyata (Observer), Kimihiro Komori (Chief director of the Japanese Society for Vascular Surgery)

NCD Vascular Surgery Data analyzers: Arata Takahashi

## Notes

This is a translation of Jpn J Vasc Surg 2020; 29: 15–31.
