# ERRATUM

**DOI:** 10.3400/avd.err.21-01001

**Published:** 2021-09-25

Vol. 13 (2020) No. 4 pp. 474–493

Vascular Surgery in Japan: 2014 Annual Report by the Japanese Society for Vascular Surgery

The Japanese Society for Vascular Surgery Database Management Committee Member, and NCD Vascular Surgery Data Analysis Team

In the above secondary publication article, numerical data and resulting errors were found after the publication. The erratum of the original Japanese article was published in Japanese Journal of Vascular Surgery Vol. 30 (2021) No. 5. The same corrections and corrected version of the table are given below.

p. 474, Left, ll. 11–12

Incorrect:

**Results:** In total 113,296 vascular treatments were registered by 1,002 institutions in 2014.

Correct:

**Results:** In total 113,515 vascular treatments were registered by 1,002 institutions in 2014.

p. 474, Left, ll. 17–19

Incorrect:

The number of vascular treatments in each field was 21,085, 14,344, 4,799, 2,088, 1,598, 42,864, and 26,518, respectively.

Correct:

The number of vascular treatments in each field was 21,085, 14,344, 4,799, 2,088, 1,817, 42,864, and 26,518, respectively.

p. 475, Left, ll. 25–28

Incorrect:

**Tabulation/Statistical Analysis Results**

In 2014, 113,296 vascular surgery cases were registered in NCD (an increase of 12.8% over the previous year), exceeding 110,000 cases.

Correct:

**Tabulation/Statistical Analysis Results**

In 2014, 113,515 vascular surgery cases were registered in NCD (an increase of 13.0% over the previous year), exceeding 110,000 cases.

p. 486, Right, ll. 15–24

Incorrect:

**1) Vascular graft infection (Table 6-1)**

We recorded 264 cases of vascular graft infection, which mostly occurred in the region classified as “other” (96.2%). It included the upper limb arteries, and there were no cases registered that involved the femoral artery-peripheral artery. Owing to the increase in cases of endovascular treatment for the lower limb arteries, it was expected that prosthetic graft revascularization would decrease. However, no cases were reported to use endovascular recanalization. The majority of other cases including those involving the upper limb arteries experienced prosthetic cutaneous fistula, of which, few were repaired by endovascular recanalization.

Correct:

**1) Vascular graft infection (Table 6-1)**

As vascular graft infection, 483 cases were registered. 52.6% of which were the others region, including the arch branching and upper limb artery. In this region, the most prevalent condition of infection is the cutaneous fistula of vascular grafts, many of which were inferred to be infection in the shunts for dialysis. 21.9% of graft infection were femoro-distal artery. The overall operative mortality was 6.8%, and in-hospital mortality was 14.3%.

p. 487, **Table 6-1**

Incorrect:

**Table:** Table 6 Revascularization for vascular complication after revascularization Table 6-1 Graft infection

Position of infected garft	Cases	Mortality		Status of infected graft				Procedure for graft infection			Material for revision or redo surgery				
		30-day mortality	Hospital mortality	Sepsis	Graft-GI fistula*^31)^	Graft-skin fistula*^31)^	Others	In-situ replacement	Extra-anatomical bypass	Others	Polyester	ePTFE	Autogenous vessel	Cryo-preserved homograft	Others
Descending thoracic aorta	5	3	3	4	0	0	1	1	0	4	1	0	0	0	0
Thoracoabdominal aorta	5	0	0	3	0	1	1	1	0	3	2	2	0	0	0
Abdominal aorta-iliac artery	0	0	0	0	0	0	0	0	0	0	0	0	0	0	0
Abdominal aorta-femoral artery	0	0	0	0	0	0	0	0	0	0	0	0	0	0	0
Femoro-distal artery	0	0	0	0	0	0	0	0	0	0	0	0	0	0	0
Others*^32)^	254	17	31	60	3	110	100	15	0	202	17	64	26	0	5
Total	264	20	34	67	3	111	102	17	0	209	20	66	26	0	5

＊31) Including anastomotic disruption. ＊32) Cases with graft infection involving aortic arch branch or upeer limb artery are listed on this column. Abbreviation; GI: gastrointestinal

Correct:

**Table:** Table 6 Revascularization for vascular complication after revascularization Table 6-1 Graft infection

Position of infected graft	Cases	Mortality		Status of infected graft				Procedure for graft infection			Material for revision or redo surgery				
		30-day mortality	Hospital mortality	Sepsis	Graft-GI fistula*^31)^	Graft-skin fistula*^31)^	Others	In-situ replacement	Extra-anatomical bypass	Others	Polyester	ePTFE	Autogenous vessel	Cryo-preserved homograft	Others
Descending thoracic aorta	5	3	3	4	0	0	1	1	0	4	1	0	0	0	0
Thoracoabdominal aorta	5	0	0	3	0	1	1	1	0	3	2	2	0	0	0
Abdominal aorta-iliac artery	59	8	18	25	22	4	16	23	0	19	26	13	5	0	5
Abdominal aorta-femoral artery	54	3	8	12	3	21	18	9	0	29	9	15	7	0	3
Femoro-distal artery	106	2	9	24	1	48	37	9	0	79	11	24	21	1	3
Others*^32)^	254	17	31	60	3	110	100	15	0	202	17	64	26	0	5
Total	483	33	69	128	29	184	173	58	0	336	66	118	59	1	16

＊31) Including anastomotic disruption. ＊32) Cases with graft infection involving aortic arch branch or upper limb artery are listed on this column. Abbreviation; GI: gastrointestinal

